# Social Factors Key to Landscape-Scale Coastal Restoration: Lessons Learned from Three U.S. Case Studies

**DOI:** 10.3390/su12030869

**Published:** 2020

**Authors:** Bryan M. DeAngelis, Ariana E. Sutton-Grier, Allison Colden, Katie K. Arkema, Christopher J. Baillie, Richard O. Bennett, Jeff Benoit, Seth Blitch, Anthony Chatwin, Alyssa Dausman, Rachel K. Gittman, Holly S. Greening, Jessica R. Henkel, Rachel Houge, Ron Howard, A. Randall Hughes, Jeremy Lowe, Steven B. Scyphers, Edward T. Sherwood, Stephanie Westby, Jonathan H. Grabowski

**Affiliations:** 1 The Nature Conservancy, URI Bay Campus, Narragansett, RI 02882, USA; 2 Earth System Science Interdisciplinary Center, University of Maryland, College Park, MD 20740, USA; 3 Chesapeake Bay Foundation, Annapolis, MD 21403, USA; 4 Natural Capital Project, Woods Institute for the Environment, Stanford University, Stanford, CA 94305, USA; 5 School of Environmental and Forest Sciences, University of Washington, Seattle, WA 98195, USA; 6 Department of Biology and Coastal Studies Institute, East Carolina University, Greenville, NC 27858, USA; 7 United States Fish and Wildlife Service, Hadley, MA 01035, USA; 8 Restore America’s Estuaries, Arlington, VA 22201, USA; 9 The Nature Conservancy, Baton Rouge, LA 70802, USA; 10 National Fish and Wildlife Foundation, Washington, DC 20005, USA; 11 The Water Institute of the Gulf, Baton Rouge, LA 70802, USA; 12 Coastwise Partners, St. Petersburg, FL 34219, USA; 13 Gulf Coast Ecosystem Restoration Council, New Orleans, LA 70130, USA; 14 United States Environmental Protection Agency Gulf of Mexico Program, Gulfport, MS 39501, USA; 15 United States Department of Agriculture, Natural Resource Conservation Service, Gulf Coast Ecosystem Restoration Team, Madison, MS 39110, USA; 16 Department of Marine and Environmental Sciences, Northeastern University, Marine Science Center, Nahant, MA 01908, USA; 17 San Francisco Estuary Institute, Richmond, CA 94804, USA; 18 Tampa Bay Estuary Program, St. Petersburg, FL 33701, USA; 19 National Oceanic and Atmospheric Administration, Restoration Center, Annapolis, MD 21401, USA

**Keywords:** coastal restoration, oyster, marsh, seagrass, restoration success, coastal habitat

## Abstract

In the United States, extensive investments have been made to restore the ecological function and services of coastal marine habitats. Despite a growing body of science supporting coastal restoration, few studies have addressed the suite of societally enabling conditions that helped facilitate successful restoration and recovery efforts that occurred at meaningful ecological (i.e., ecosystem) scales, and where restoration efforts were sustained for longer (i.e., several years to decades) periods. Here, we examined three case studies involving large-scale and long-term restoration efforts including the seagrass restoration effort in Tampa Bay, Florida, the oyster restoration effort in the Chesapeake Bay in Maryland and Virginia, and the tidal marsh restoration effort in San Francisco Bay, California. The ecological systems and the specifics of the ecological restoration were not the focus of our study. Rather, we focused on the underlying social and political contexts of each case study and found common themes of the factors of restoration which appear to be important for maintaining support for large-scale restoration efforts. Four critical elements for sustaining public and/or political support for large-scale restoration include: (1) resources should be invested in building public support prior to significant investments into ecological restoration; (2) building political support provides a level of significance to the recovery planning efforts and creates motivation to set and achieve meaningful recovery goals; (3) recovery plans need to be science-based with clear, measurable goals that resonate with the public; and (4) the accountability of progress toward reaching goals needs to be communicated frequently and in a way that the general public comprehends. These conclusions may help other communities move away from repetitive, single, and seemingly unconnected restoration projects towards more large-scale, bigger impact, and coordinated restoration efforts.

## 1. Introduction

Throughout the United States, extensive investments have been made to restore lost ecological functions and services resulting from habitat loss and degradation. The restoration of coastal marine habitats, such as salt marshes, submerged aquatic vegetation, oyster reefs, mangroves, and corals, has occurred in every coastal state and U.S. territory. Coastal restoration has increased in terms of both number and scale of projects over the past decade, yet many restoration projects are still small relative to the degree of habitat loss that has occurred over the past two centuries [1,2]. This restoration lag is likely due to many factors including the lack of suitable area for projects, the cost of habitat restoration, and the availability of funding [3,4]. Furthermore, many restoration projects are implemented with minimal acknowledgement or understanding of how an individual restoration project contributes to ecosystem-scale (e.g., bay or estuary-wide) functioning or regional management goals [5]. The lack of funding for long-term monitoring of restoration projects further reduces the ability to disentangle the degree to which these activities help recover ecosystem functioning.

There have been several excellent academic reviews that have addressed and emphasized the ecological theory that must be considered when developing recovery plans (e.g., ecological baselines, stable and unstable ecological states, setting quantitative restoration objectives). These contributions to the literature have been paramount in providing restoration practitioners with a better understanding of the science underpinning ecological restoration and recovery, and the importance of advancing that science (e.g., [6–18] and others). There have been historically fewer reviews, however, that have addressed the suite of societally enabling conditions that existed in ecosystem-scale projects where coastal restoration efforts were sustained for longer periods. This may be in part because large-scale restoration efforts are relatively rare. However, it may also be because most of the initial focus of coastal ecosystem restoration research has been on understanding the ecological processes and outcomes of restoration, while there has been less focus on the social factors important to coastal restoration. Specifically, there has been little research examining what societal factors are important to maintain public and/or political support for large-scale restoration, even though this is a major potential barrier to ecosystem recovery.

To better understand the human and societal conditions that lead to successful coastal restoration and ecosystem recovery, we reviewed three case studies involving large-scale coastal restoration efforts and determined whether there are common principles for sustaining support for these large efforts that can guide future efforts. The case studies are the seagrass restoration effort in Tampa Bay (TB), Florida, the oyster restoration effort in the Chesapeake Bay (CB) in Maryland and Virginia, and the tidal marsh restoration effort in San Francisco Bay (SFB). While each case is geographically and ecologically different, we focused on the societal commonalities across the three case studies that point to important social factors that are needed to facilitate coastal ecosystem restoration and recovery. Furthermore, we explored the important roles of different stakeholder groups, including citizens, governments and politicians, and scientists.

All three case studies demonstrate the potential of coordinated, large-scale restoration efforts to achieve landscape-scale conservation goals. Based on lessons from these case studies, we draw conclusions that may help other communities move away from repetitive, single, and seemingly unconnected restoration projects towards more large-scale, bigger impact, societally-supported and coordinated restoration efforts.

## 2. Materials and Methods

### 2.1. Selecting Case Studies

**Figure 1.**
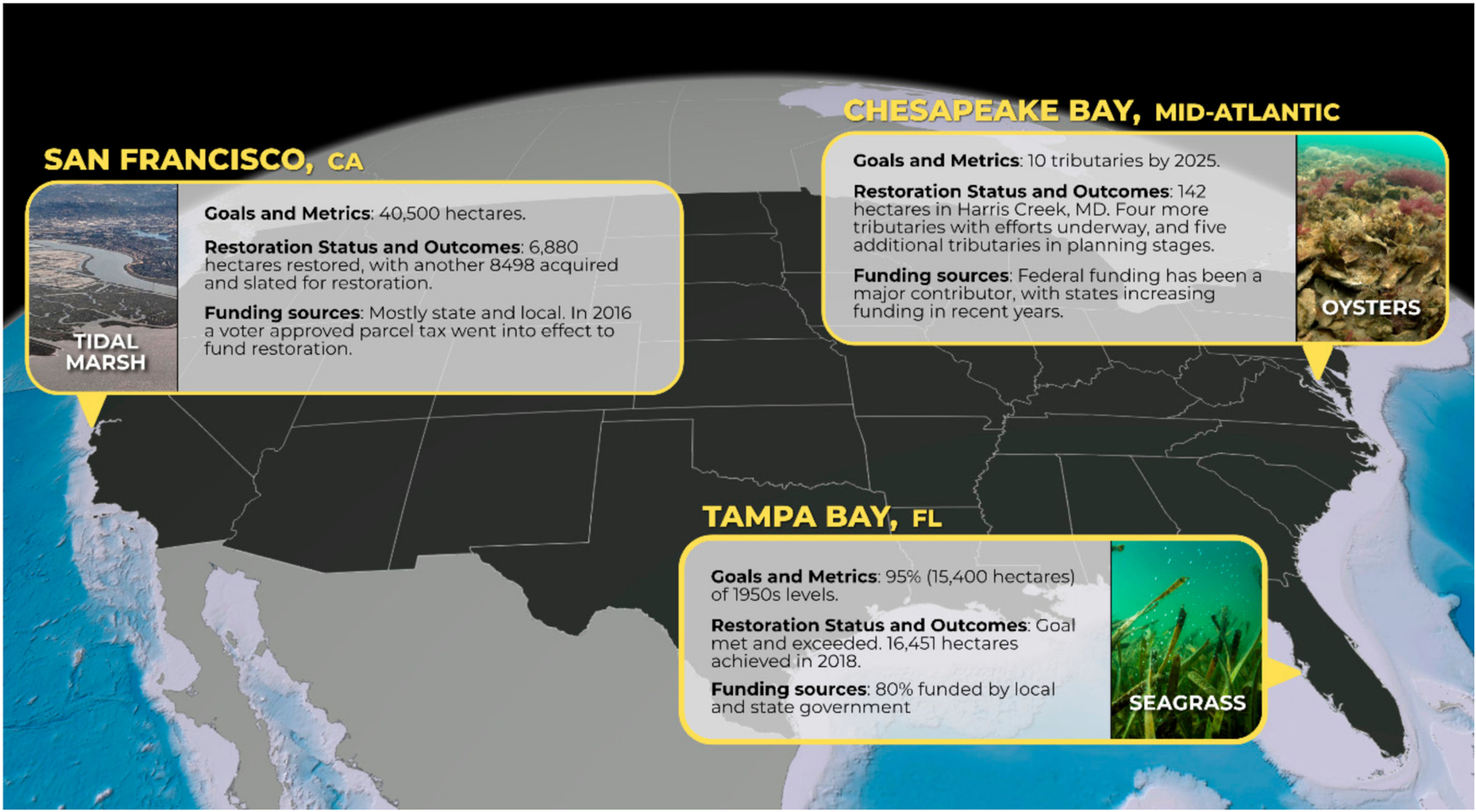
Infographic of summary Goals and Metrics, Restoration Status and Outcomes, and Funding Sources for three case study locations. Tampa Bay: Photo Credit, James R. White. Restoration focused on rehabilitation of seagrasses via improvements in water quality, but also to restore four other key habitats to the proportion they were in the 1950s relative to seagrasses. Other aquatic habitats like mangroves are at or near this goal, and some are increasing in extent. Funding has averaged USD 250M per year. Chesapeake Bay: Photo Credit, Oyster Recovery Partnership. Goals were based on “Oyster Success Metrics” defining reef- and landscape-level criteria necessary for a tributary to be considered “restored”. The 142 hectares restored in Harris Creek is presently the largest oyster reef restoration project in the world. Since 2011, more than USD 51M of federal dollars has been spent on oyster restoration in MD alone. San Francisco Bay: Photo Credit, Dicklyon. The 40,500 hectares recommended by the Goals Project was based around improved habitat quality and quantity to support key species and presented at various geographic scales. In 2002, voters approved USD 200M to implement projects recommended in the Goals Project report. The 2016 voter-approved parcel tax is expected to raise USD 25M annually for restoration.

We developed an initial list of potential landscape-scale restoration case studies around the U.S. using the following criteria: (1) the restoration had to be either completed or with enough active project implementation completed to assess the degree of restoration; (2) the case had to be at a geographic scale that was larger than the singular project level, and involve substantial regional and local coordination to implement it; (3) there had to be enough information available on the restoration efforts to develop a comprehensive case study; and (4) the list needed to represent multiple regions around the U.S. and a diversity of restored coastal habitat types to avoid developing generalities that could potentially be specific to one region or habitat type. To create an initial list of candidate cases that met the criteria above, we first consulted an expert coastal restoration working group of more than a dozen federal, academic, and non-governmental organization (NGO) professionals in coastal restoration. Using the initial list created by the working group, we selected 9 potential cases to query for additional information (See Table S1 in Supplemental Materials). To collect information on those 9 cases in a standardized manner, we created a questionnaire with eight questions which we sent directly to specific local experts who were familiar with each case (See Questionnaire in Supplementary Materials). The questionnaire resulted in the collection of qualitative information on each of the candidate cases. The questionnaire included questions on the goals of the restoration efforts (e.g., output or outcome based); whether the restoration was singular or multi-habitat based; the geographic scope of the restoration efforts; the level of participation from partners and other stakeholders in the restoration planning phase; the status of the past and current restoration; information on funding; and the level of public awareness of the restoration efforts. Based on the questionnaire responses, we selected the seagrass restoration effort in Tampa Bay (TB), Florida, the oyster restoration effort in the Chesapeake Bay (CB) in Maryland and Virginia, and the tidal marsh restoration effort in San Francisco Bay (SFB), California (Figure 1).

### 2.2. Reviewing Cases

To review each case, we mined the peer-reviewed and gray literature for information and reviewed any management plans developed for the case. We also conducted interviews with local experts, particularly those who were involved with the development of the restoration plans for each case. We gathered information specifically about four topics: (1) the background, history and ecological context of the geographic area; (2) a history of the restoration plan (i.e., how and why it was developed, and the restoration goals); (3) the status, results, and impacts of the restoration; and (4) the role of stakeholder involvement, including resource management and funding, in the restoration.

## 3. Results

### 3.1. Tampa Bay, Florida

#### 3.1.1. Background and Ecological Context

Tampa Bay (TB), Florida is arguably one of the United States’ greatest success stories regarding ecosystem restoration, and it is recognized internationally for its remarkable progress towards recovery [19–27]. TB is a relatively large (water surface area of 1031 km^2^) embayment on the west coast of Florida with a watershed of approximately 5700 km^2^ [24,28]. The subtropical estuary primarily includes seagrass meadows, emergent tidal wetlands (mangroves, salt marshes, salt barrens), tidal flats, and oyster reefs/bars [29]. Population growth has put pressure on these coastal ecosystems since the 1880s. By 1980, urban development activities (e.g., poorly treated wastewater, port channel dredging, and shoreline dredge and fill) had negatively impacted coastal wetlands and seagrass beds [28,30,31]. By the early 1980’s 44% of emergent wetlands and 81% of seagrass areal extent were lost [32]. Circulation and salinity patterns were changed, and nutrient pollution had so degraded water quality by 1980 that many considered the bay to be “dead” [30].

#### 3.1.2. Restoration Plan at Scale: History, Development, and Goals

Citizens of TB demanded action [22,25] in the 1980’s, and as a result, legislation was enacted requiring more stringent treatment standards for wastewater plants discharging to TB. Recognizing the need for a comprehensive bay restoration and protection plan, the Tampa Bay National Estuary Program (TBNEP) was established in 1991 to address the harmful effects of population growth and coastal development on the water quality and coastal wetlands of TB. National Estuary Programs are place-based Environmental Protection Agency (EPA)-funded programs that use federal dollars to leverage additional funding and partner support (EPA National Estuary Program website). The TBNEP was responsible for the development and implementation of a science-based management and restoration plan for the TB estuary and leveraged an interlocal funding agreement to become the Tampa Bay Estuary Program (TBEP) in 1998 [31]. The TBEP helped coordinate and oversee organizing technical efforts to develop goals for restoring the estuary, but the impetus to implement projects fostering ecological change was from the community via considerable citizen input and pressure from both public and private entities and stakeholders [25]. The TBEP developed a Comprehensive Conservation and Management Plan in December 1996—subsequently updated in 2006 and 2017 [29]—that included measurable goals for the achievement of the Bay’s designated uses and to support full aquatic life protection by identifying a diverse set of actions and strategies to improve environmental quality [22].

For TB, seagrasses are the “canary in the coal mine”, as much of the focus of the recovery efforts revolved around meeting water quality goals that promote seagrass recovery. Seagrass recovery goals were established from aerial photography of the 1950s (a period prior to major development impacts), and a TBEP Policy Board decision to restore the Bay to 95% of its 1950s seagrass acreage. To achieve this goal, empirical analyses were used to derive nitrogen-loading targets sufficient to maintain water quality requirements of *Thalassia testudinum* [21]. For four other key habitats (mangroves, salt marsh, freshwater wetlands and salt barrens), quantifiable restoration and protection targets were set by calculating the relative proportion of each of these habitats in comparison to their original amounts in the 1950s [31]. As such, the recovery of TB is often not referred to as “restoration”, but rather “rehabilitation”, given the acknowledgment that returning to a state prior to significant anthropogenic impact is neither feasible nor attainable [33]. This concept, termed “Restoring the Balance,” had broad appeal to both the TB public and resource managers [22].

#### 3.1.3. Restoration Plan Status and Outcomes

TB is considered a worldwide model for estuary recovery. As of the 2018 assessment [34], the bay-wide seagrass recovery goal of 15,378 hectares (38,000 acres) was surpassed with an estimated 16,451 hectares (40,652 acres). Likewise, other important estuarine habitats, like mangroves, are increasing in extent [35].

Several reviews of the TB recovery efforts have identified the development of quantitative restoration and recovery goals as being a critical component of the overall recovery movement because they allowed collective agreement on a clear path forward to achieve a ‘healthier’ Tampa Bay, thereby bringing everyone together around those common goals [21–23,25,36]. It also enabled the TBEP to relay positive progress towards clear benchmarks of water quality and ecosystem recovery, which further fostered community buy-in and momentum for continuing the investments and commitments to nutrient-load reduction projects that would help toward the goal.

#### 3.1.4. Role of Stakeholder Involvement

The TBEP concluded that establishing quantitative goals early in the process resulted in meaningful participation by local stakeholders, as evidenced by their voluntary participation in the comprehensive nutrient management strategy for TB [25]. Citizen and stakeholder involvement have been a critical component to meeting seagrass recovery goals in TB. Initial state regulations implemented in the 1980’s requiring wastewater treatment facilities to significantly reduce nutrient discharges were a direct result of citizens’ call for action. Again, in the early 1990’s as part of the TBEP’s development of a comprehensive restoration plan for Tampa Bay, citizens identified improving water quality, fishing, and swimming conditions as primary recovery goals. This support ultimately led to the development of specific, numeric water quality targets and seagrass restoration goals for the Bay. Furthermore, implementing the actions set forth in the recovery plans required broad partnerships and collaborative projects among scientists, resource managers, citizens, and public agencies to collectively achieve the environmental and economic benefits currently realized from a ‘healthy’ Tampa Bay [29].

On-the-ground habitat restoration has only been one component of the suite of ecological restoration activities conducted in TB. Diverse habitat protection and management activities have been pursued by local and regional entities throughout the estuary’s watershed. Other work implemented to meet the TB’s recovery goals revolved around infrastructure modifications and improvements, or best management practice implementation, primarily focused on directly reducing atmospheric or stormwater sources of nitrogen inputs to the Bay. From 1990–2017, more than 450 nutrient load reduction projects have been completed, ranging from municipal wastewater treatment facility upgrades to residential, agricultural, and urban storm water runoff reduction projects, improvements in fertilizer manufacturing and shipping activities, and pet waste reduction campaigns in neighborhoods and parks [37,38].

#### 3.1.5. Funding

According to Russel and Greening [26], public agencies contributed approximately USD 250 million per year across nine different program areas corresponding to TB resource management priorities, including pollution control, wastewater and storm-water management, living resources, habitat preservation and restoration, land acquisition, dredged material management, regulation and enforcement, public awareness, and administration planning and coordination. The TBEP estimates that approximately 80% was funded from local or state sources, and while there were some federal grants, they summed to a relatively small percentage in comparison to regional investments by the Southwest Florida Water Management District and other local governments [33]. The role of the TBEP, however, cannot be over-stated. While the TBEP is not a large, direct contributor of funding for infrastructure and restoration activities contributing to bay wide water quality improvements, their scientific, advisory, and coordination efforts underpinned and helped garner the necessary community support needed to rally around a shared recovery goal for TB. For example, TBEP is a neutral facilitator and convener of the public/private Tampa Bay Nitrogen Management Consortium (TBNMC), an alliance of more than 45 local governments, regulatory agencies, key industries, and utilities formed to work collaboratively to meet nitrogen management targets supportive of seagrass recovery goals. The TBNMC has contributed more than USD 0.7 billion since the mid 1990’s on various nitrogen load reduction projects [29]. The degree of organization, coordination and collaboration necessary to initiate and maintain the many restoration activities being conducted in the TB estuary and its watershed would have been extremely difficult without federal, state and local government commitment, and funding to support TBEP’s role.

### 3.2. Chesapeake Bay, Maryland and Virginia

#### 3.2.1. Background and Ecological Context

The Chesapeake Bay (CB) is the largest estuary in the United States, with a watershed of 165,800 km^2^ that spans six states. There are more than 150 major rivers in the watershed, but roughly 80% of the freshwater input to the CB comes from the Susquehanna, Potomac, and James Rivers. The estuary is relatively shallow, averaging 6.5 m deep, with a deeper channel (20–30 m) running through the main stem. CB consists of many habitats such as tidal marshes, seagrass beds, oyster reefs, hard bottom, and mud flats [39].

With a watershed area roughly fourteen times the surface area of the estuary [39], land use has had a profound influence on the productivity and structure of the CB ecosystem, which has changed significantly in the past 200 years [40,41]. Human settlement in the early 17th century was followed by rapid deforestation that increased nutrient and sediment loading to the system, with particularly negative impacts to oyster reefs [40,42–44]. As nutrient loading increased, oysters initially benefited from greater primary productivity, but continued eutrophication led to persistent seasonal hypoxia and the silting over of the remnant oyster reefs [42,43]. Water quality issues were exacerbated by the overharvesting of oysters, which reduced the yield per recruit to 8.4% of the unfished population [44] and further worsened water quality by reducing filtration capacity [45,46]. By the 1950s, it was evident that the system had exceeded a water quality tipping point, leading to a rapid decline in several important coastal habitats (seagrasses, saltmarshes, oyster reefs [47,48]), including a 99.7% decline in oyster abundance in the Upper Chesapeake Bay since the early 1800’s [49]. Cumulative economic losses of more than $4 billion over the past three decades have affected the coastal communities of Maryland and Virginia due to loss of oyster harvest revenue and impacts to associated industries [50]. Unquantified losses of ecosystem services other than extractive value and related industries are likely much higher [51].

#### 3.2.2. Restoration Plan at Scale: History, Development, and Goals

In light of deteriorating water quality and ecosystem impacts, citizens appealed to elected officials to take action. A key development in the Bay clean-up process was when Senator Charles “Mac” Mathias from Maryland responded to citizens’ appeals by commissioning a 5-year, USD 27 million study to pinpoint the causes of the Chesapeake’s problems. This study led to the development of the EPA’s Chesapeake Bay Program and the first Chesapeake Bay Agreement, signed in 1983 [52]. The agreement consisted of a simple one-page pledge by the Governors of Maryland, Pennsylvania, and Virginia, along with representatives from Washington, D.C., the EPA, and the Chesapeake Bay Commission, to work together to restore the health of CB. Following this initial effort, the Chesapeake 2000 Agreement was the first to set a quantitative oyster restoration goal—to increase the oyster population in the Bay ten-fold by 2010. Yet, even this ambitious goal failed to produce a significant improvement in oyster populations, as the agreement lacked a specific implementation plan [53] and surveys of the oyster population were inadequate to determine progress towards the ten-fold population goal [54].

In 2009, President Obama issued Executive Order 13508, which instructed federal agencies to develop a coordinated federal strategy for the restoration and protection of CB, including its oyster populations, within 180 days of its issuance [55]. This directive resulted in the Strategy for Protecting and Restoring the CB Watershed [56], which established the goal of restoring the oyster populations of 20 CB tributaries by 2025. This was the first quantifiable goal that focused on large-scale restoration. Through this directive and the resulting goal, the region was able to quickly galvanize the technical expertise, funding, and coordination of federal efforts to begin addressing this large-scale coordinated effort [57].

In 2011, restoration partners came together to define a priori metrics, through consultation with external oyster scientists, that would define restoration success. The “Oyster Success Metrics,” developed in 2011, defined reef- and landscape-level criteria necessary for a tributary to be considered “restored” [58]. From these metrics, restoration partners and scientists worked backward to determine the restoration effort in each area that would most likely achieve target oyster densities, biomass, and reef acreage as well as the necessary monitoring protocols for assessing if targets were met.

The 2014 CB Watershed Agreement solidified state and federal partners’ commitments to large-scale oyster restoration in ten tributaries by 2025, a revised goal that more accurately reflected the feasibility of the project. The “10 tributaries by 2025” goal is the primary driver for current oyster restoration efforts in CB.

#### 3.2.3. Restoration Plan Status and Outcomes

On-the-ground work to implement tributary-scale oyster restoration began in 2011. By 2016, construction on the first restoration tributary, Harris Creek, in Maryland, and the largest oyster restoration project in the world to date, was complete, resulting in the restoration or enhancement of 142 hectares (350 acres) of oyster reef habitat [59]. Restoration activities are currently underway in four other CB tributaries, and all 10 tributaries have been at least tentatively selected and are in the survey and planning phase [60].

The ability to track and report progress toward the 10-tributary restoration goal has helped to enhance public support for the project. A bipartisan opinion poll conducted in February 2018 indicated that 83% of Maryland voters support tributary-scale oyster restoration in the state [61].

Although the Oyster Success Metrics focus on quantitative outputs (e.g., area restored, oyster density), they are linked to ecosystem outcomes through additional criteria, including multiple oyster age classes and reef footprint and accretion. These metrics are intended as a quantitative proxy for ecosystem services (e.g., fish and macrofauna habitat provisioning, water quality improvements) not directly measured through the monitoring program [58]. Additional research programs spurred by the large-scale restoration goal are working to directly assess the ecosystem service benefits of large-scale oyster restoration. Thus far, results of these studies have indicated that large-scale oyster restoration will significantly increase blue crab (*Callinectes sapdius*) biomass, thereby benefitting associated blue crab fisheries [62]. Significant advancements in quantifying denitrification on restored oyster reefs have also led to the approval of oyster aquaculture as an in-water best management practice for nitrogen and phosphorus removal by the EPA [63].

#### 3.2.4. Role of Stakeholder Involvement

The restoration efforts in CB are unique, as the governance structure of the Chesapeake Bay Program leads to a primarily top-down approach where most of the coordination and funding occurs at the federal level [64]. This approach is appropriate for CB, where efforts are multi-jurisdictional and require cooperation amongst multiple states to achieve a common objective [65]. While federal agencies are responsible for coordination, oyster restoration requires full support from the states as restoration work is occurring in waters under their jurisdiction. Thus, states, along with local governments, watershed groups, and other relevant stakeholder groups, are full partners in these efforts, both financially and logistically [65]. Additionally, each of the outcomes of the Chesapeake Bay Watershed Agreement is assigned to a Goal Implementation Team, which consists of federal and state agency partners along with consulting scientists and local stakeholders, such as local watershed associations [65]. Through these Teams, local and regional interests are given a forum through which to contribute to restoration planning and policy.

#### 3.2.5. Funding

Executive Order 13,508 provided a clear, common goal around which federal and state agencies could target restoration work. Though several agencies, particularly the Army Corps of Engineers and NOAA, had already been engaged in oyster restoration in Chesapeake Bay, setting large-scale targets for restoration necessitated the cooperation and coordination of state and federal agencies to achieve the funding levels required to achieve these goals. Through the mechanism of federal-state cost-share agreements, federal dollars were leveraged with state funding, usually at a ratio of 75% federal and 25% state, though the funding arrangements differed by agency and some did not require state matching funds. Additionally, tributaries in which large-scale oyster restoration is conducted are protected from harvest through statute or regulation. This assurance of protection has resulted in positive feedbacks that have bolstered larger restoration efforts. For instance, it has catalyzed public–private partnerships such as the Chesapeake Bay Stewardship Fund [66] that brought corporate philanthropy to oyster restoration, and invited further investment from watershed organizations and community groups interested in contributing to areas closed to harvest.

### 3.3. San Francisco Bay, California

#### 3.3.1. Background and Ecological Context

The San Francisco Bay (SFB), together with the upstream inland Delta, comprises the largest estuary (~4000 km^2^) on the U.S. Pacific Coast, and remains one of California’s most important ecosystems. The evolution of the SFB involves a complicated history of natural and human-induced factors including sediment ebbs and flows, sea level changes, diking, and development [67–69]. Prior to the mid-19th century, the SFB and the inland Delta were comprised of approximately 1300 km^2^ of open water and another 2200 km^2^ of fresh-, brackish- and salt-water marsh [70,71]. The region was heavily modified by humans to support a rapidly growing population with the gold rush of the 1800’s, including diking wetlands for agricultural land [71]. Simultaneously, gold-seekers were perfecting hydraulic mining where high-pressure streams of water led to destruction of the hills and flushing of a great deal of sediment into the rivers and creeks, delivering nearly a billion cubic meters of sediments between 1849–1914 [72]. By 1930, most of the of freshwater marshes were diked and farmed, and 80% of the Bay’s salt marshes and intertidal mudflats were turned into salt ponds, cow pastures, or residential and commercial real estate [71], and the Bay was continually being filled to provide more space for ports, industry, garbage dumps and other development well into the 1960s. The result of the anthropogenic pressures on SFB was the loss of wildlife habitats and a reduction in tide-related flushing, which in turn has led to progressive deterioration of the Bay’s water quality [67–69,71].

#### 3.3.2. Restoration Plan at Scale: History, Development, and Goals

There was a growing public concern for the health of the Bay, and in 1961 three women—Silvia McLaughlin, Catherine “Kay” Kerr, and Esther Gulick—took action against the filling of the Bay to create the association that is now known as Save the Bay [73]. At Save the Bay’s urging, the McAteer-Petris Act was enacted in 1965, serving as the key legal provision preventing the indiscriminate filling of the Bay, and establishing the San Francisco Bay Conservation Development Commission (BCDC)—the world’s first coastal protection agency [74]. The BCDC was the first agency set up to look at the Bay as a whole system, a switch from the previous management, where municipalities only considered their own parts of the Bay. While the primary mission of the BCDC is to protect the Bay, in 1987 the EPA, as part of its National Estuary Program, established the San Francisco Estuary Project (SFEP), with the mission of restoring the health of the Bay’s ecosystem. Bringing together the environmental community, private sector and government, the SFEP was a collaborative effort that focused much-needed attention on the San Francisco Estuary [75]. In addition to identifying the Estuary’s most critical problems, a major project of the SFEP was a Comprehensive Conservation and Management Plan (CCMP) [75], which was signed by the Governor of California and the Administrator of the U.S. EPA in 1993, and was then updated in 2007 and 2016. The CCMP identified 145 actions necessary to “restore and maintain the estuary’s chemical, physical, and biological integrity”, as well as specifying the creation of an estuary-wide plan to “protect, enhance, restore, and create wetlands in the Estuary”, and that this plan will be based on habitat goals designed to protect wildlife [75].

By 1995, a large group of Bay scientists and resource managers, including nine state and federal agencies, came together to develop a “shared vision” for habitat change in the whole estuary. This effort was called the San Francisco Bay Area Wetlands Ecosystem Goals Project (covering Suisan Bay to the South Bay) [70]. The 1999 report was later updated in 2015 [76] to address the projected effects of climate change. While the acreage goals of the 1999 report remained the same, the 2015 update synthesized the latest science, and incorporated projected changes through 2100 to generate new recommendations for achieving a healthy ecosystem. The focus of the Goals Project is based around improved habitat quality and quantity to support key species. In addition to wildlife being specified in the CCMP, this decision was justified because concern about species and human health drives most federal and state environmental laws and policies. Furthermore, they surmised that protecting key species by improving their habitats would concurrently improve other important wetland functions [70].

The approach for developing the habitat goals involved several iterative steps that included more than 65 qualified experts. Five focus teams were developed for plants, fish, and wildlife. The focus teams developed lists of key species and identified their habitat requirements. Seven key habitats were identified within the baylands, and seven key habitats were identified outside of the baylands but within the baylands ecosystem. The project next mapped the historic and current habitat area of each. The focus teams blended the habitat recommendations into a conceptual vision that balanced the competing needs of the many baylands species. Ultimately, this two-year process allowed them to calculate area for each of the key habitats and compared the proposed future habitat area to the historic and modern amounts [70].

The outcome of these efforts resulted in specific habitat goal recommendations, presented in terms of area, that were required to support key species. The habitat goals were presented at various geographic scales, including recommendations for four main subregions, as well as for segments of each subregion. Notably, the regional area goals called for tidal marsh restoration on an unprecedented scale: 24,281 hectares (60,000 acres) to be restored, to reach a total of 40,466 hectares (100,000 acres). Setting goals to restore this degree of salt marsh required anticipated reductions of other associated habitats (e.g., salt ponds); thus, the report suggested offsetting the reductions by maximizing wildlife management effectiveness in those associated habitats, thereby still increasing the region’s overall ability to support shorebirds, waterfowl, mammals, and other wildlife [70]. The 2015 science update to the original Goals report implemented adaptive management and improved upon the original 1999 goals. The update addressed issues arising since 1999 such as climate change and reduction in sediment supply. It also built on 15 years of landscape-scale restoration experience, ultimately adapting the Goals to reflect increased knowledge and science since the original report.

#### 3.3.3. Restoration Plan Status and Outcomes

Prior to the publication of the Goals Project, tidal wetland restoration projects were few and relatively small in scale, with the largest around 350 acres [76,77]. By providing a consensus-based scientific vision of the kinds, amounts, and distribution of habitats needed to sustain healthy populations of fish and wildlife for the entire region, the Goals Project gave regulators, resource managers, and citizens the framework necessary to pursue large-scale restoration for bay habitats. For example, the South Bay Salt Pond Restoration Project is the largest tidal wetland project on the US West Coast, the footprint of which encompasses nearly the entirety of the southern end of the Bay, and will restore 6111 hectares (15,100 acres) when complete [78]. Nineteen years after the Goals Project report was published, 6880 hectares (17,000 acres) of wetland habitat have been restored, and another 8498 hectares (21,000 acres) of diked baylands has been acquired and slated for restoration to tidal marsh and associated habitats [79]. Beyond setting the quantitative goals for restoration, the Goals Project provides guidance to coordinate the restoration and acquisition investments, ensuring the projects and land acquisitions are best suited to achieve landscape-scale benefits for the entire Bay system.

#### 3.3.4. Role of Stakeholder Involvement

The SFB is arguably one of the greatest stories of how stakeholder involvement, particularly from community members, played a pivotal role in ecosystem recovery. The story of three women, and the role they played in “saving the Bay”, is practically folklore in the region. Their actions not only created one of the most well-known conservation organizations in the U.S., but it helped kick-start a series of actions that ultimately led to a significant change in how the ecology and ecosystem of the Bay were viewed and managed. According to experts, one of the most significant outcomes was the entire stakeholder community “getting on the same page” in terms of aligning and focusing efforts on a common set of goals [79].

The approach for developing the Goals Project involved several steps, following a designed organizational structure that included stakeholder involvement throughout the process. This included a steering committee of representatives from multiple resource management and science organizations. Focus teams were developed that consisted of more than 65 science contributors, selected to participate in collaborative workgroups. An independent science review panel was created to review the draft Goals. Throughout the development of the Report, public outreach was extensive. The public outreach meetings provided many benefits to the process, including developing a better sense of the issues of concern, improving technical products, and ideas on how to present the Goals in a way that would make them most useful [70]. The process for the 2015 science update included a steering committee of representatives from resource management and science organizations; collaborative and open participation by science contributors organized into workgroups; an independent science review panel; and a core administrative team, including the science coordinator [76].

#### 3.3.5. Funding

The inclusion of specific, quantitative recommendations (i.e., reestablishing 100,000 acres of tidal wetlands) in the Goals Project has been integral to leveraging new funding sources for restoration. Indeed, after the Goals Project was released, funding for baylands restoration projects increased appreciably. Indicative of its importance, in 2002, the Goals Project was explicitly cited in Proposition 50, a proposition approved by voters that allocated the Wildlife Conservation Board up to USD 200 million for the implementation of restoration projects mentioned in the report. Importantly, the Goals Project has also benefitted many smaller bay restoration projects, as both state and federal agencies have increasingly used its science-based guidance to identify restoration and conservation projects that address grant program mandated habitat and water-quality enhancement objectives. In a recent historic vote, the people of the Bay Area leveed upon themselves the first regional parcel tax measure in California’s history, which will raise USD 25 million annually, resulting in USD 500 million over twenty years (Measure AA) [80].

The report and its update have become a cornerstone of policy, planning, coordination, and advocacy for the acquisition, protection, and restoration of the SFB baylands. Many public agencies have incorporated the Goals Project into regional planning and policy documents. The Goals Project has also spurred regional entities in working with members of the US House and Senate to seek a federal funding program (e.g., the San Francisco Bay Improvement Act of 2010, the San Francisco Bay Restoration Act of 2015, the San Francisco Bay Restoration Act of 2019) comparable to other nationally significant bay-restoration programs to accelerate the restoration of the bay [76].

## 4. Discussion

The three case studies differ in their geographies and the species and ecosystems being restored, but we observed similar themes among them that point to important social factors of effective landscape-scale ecosystem restoration and recovery efforts. Here, we examine each of these themes in more detail and provide an insight into the significance of each in the ability of the three case studies to achieve sustained and coordinated landscape-scale ecosystem restoration.

### 4.1. Recognizable Ecological Crisis with Public Demand for Action

In each of the cases reviewed, ecosystem degradation was well-documented by the scientific community and recognized and considered to be at a point of crisis by the public. Identifying the processes leading to degradation or decline of a natural system has been proposed as the initial step of a restoration process [5,9,10]. While this is a key step, scientific understanding of declining ecological conditions may not be enough to motivate large-scale restoration efforts. In the three cases reviewed here, not only was the decline well-documented, but there was also a corresponding public demand for action that resulted from the communities’ awareness of that decline. In each case, the strident public outcry led to political intervention which then resulted in actual restoration action. These examples highlight the importance of the public understanding the extent and consequences of the environmental crisis (e.g., ecological, societal, economic, cultural). In cases where the appropriate level of public support and demand for change does not yet exist, building public motivation may be an important first step [81], even prior to the dedicating of resources to active ecological restoration.

### 4.2. Political Response Catalyzing the Development of Estuary-Level Recovery Plans

In an excellent review of the role of ecological restoration in the turn of the millennium, Hobbs and Harris [5] suggested that political opportunism often is more critical in setting restoration priorities than any rational process. The cases we reviewed provide examples of where political support catalyzed the development of recovery plans and “set the tone” for recovery efforts.

The political support that arose from public outcry for action, and its catalytic role in developing estuary-level plans for ecological recovery, was an important commonality in each of the cases evaluated. Furthermore, in all three cases, financial and/or political support from the EPA was a fundamental component. The significance of politically-motivated calls for comprehensive recovery plans should not be underestimated. Furthermore, in all three cases there was federal expertise and coordination provided to support the recovery planning efforts. In TB, the TBEP, which was federally funded, provided the coordination and catalyst that facilitated both public and private investment in the restoration. In San Francisco, the EPA was instrumental in the development of the SFEP, and later many federal agencies were part of the effort to design the restoration goals and contributed a great deal of expertise that helped the project succeed. Further, in the CB, federal involvement was explicitly directed in Executive Order 13508, which called on seven federal agencies to work on what became the “Strategy for Protecting and Restoring the Chesapeake Bay Watershed”. Federal partners bring considerable nationally gained expertise, knowledge, and a capacity that can be critical in helping guide the comprehensive recovery planning efforts.

### 4.3. Development of Science-Based, Estuary-Level, Comprehensive Plans for Action with Clear, Measurable Goals

The concept of setting restoration goals is not new and has been addressed multiple times in the academic literature (e.g., [5–7,9,16–18,82,83] and others). Ehrenfeld [7] declared that there is no one paradigm or context for setting restoration goals. The cases reviewed here support that statement, as each went through entirely different processes to develop the recovery plan that resulted in entirely different recovery goals. Arguably, however, developing the vision for recovery—that was both founded/grounded in science and supported by the community—was the most critical element of the recovery plans.

Several key similarities amongst the three recovery plans goals may have led to their sustained success: First, none of the cases set restoration and recovery goals solely based on returning to an historic benchmark. It has been well documented that over-dependency on historical baselines as restoration goals is often unrealistic or unachievable (e.g., [6,9,14–16]). In Tampa Bay, while the seagrass restoration goals were based around a historical extent, the restoration goals of several other key habitats focused on recovering the proportions of the habitat present during an earlier, less-disturbed, period. In San Francisco, a similar approach was based on evaluating the habitat needs of targeted species, with a guiding principle of increasing the quantity and quality of wetlands without trying to “reach” the past. While in Chesapeake Bay, the aspirational oyster goal of ten tributaries restored over a 10-year horizon reflected anticipated resources required to achieve those goals (See Figure 1).

Second, each of the recovery plans were translated into quantifiable management goals that were easily understood by the public, with specific targets that enabled the clear communication of progress on restoration goals. Establishing measurable goals is critical to maximizing the chances of obtaining and demonstrating restoration success [16,84]. Furthermore, the goals should be easily observable by the public [10,84]. Thus, while recovery goals can and should be based on a range of outcomes and trajectories (e.g., [6,7,15,16] and many others), they simultaneously need to be translated into terms that the public can understand and witness progress towards. The area to be restored (e.g., acres) is often used because it is a tangible metric that is easily communicated to the public. However, goals should reflect the primary motivation for the restoration, and the service the community is seeking to “get back” from restoration of that habitat. For example, in SFB, the “goals” are framed in acres restored; however, the area-based goal is a function of the ecological service to benefit key wildlife outcomes. Despite the differences in the restoration planning process, including the community participation in it, the planning process was a critical theme in each of these cases. For example, TBEP recognized that goals needed to be framed in a manner that could be easily and convincingly communicated to the public. Changes to habitat landscapes over time are a visible and intuitive aspect of estuaries that the public can easily see, understand, and relate to. In CB, the goal of ten tributaries over ten years is easy for the public to comprehend, even if the specific ecologic metrics that define “restored” were painstakingly developed [58].

Finally, restoration goals were established in all three regions at appropriate spatial and temporal scales and with realistic recovery time-scales in mind. Longer-term (decadal) restoration trajectories that are less predictable, but more representative of real system attributes, are more realistic to accommodate variability [14]. Spatially, recovery plans need to set a trajectory that can be accomplished through the implementation of several smaller projects. In other words, it is unrealistic to expect “large-scale” to always mean bigger individual projects, since projects are often limited by funding, the amount of land available, or other factors. The role of the recovery plan is to ensure that smaller-scale projects are connected ecologically. For example, in CB, recovery goals were set to achieve a restoration of 50%–100% of the restorable bottom in each identified tributary. Those goals will be accomplished via several smaller projects that all contribute to the overall goal. The role of the science is to ensure the planning, prioritization, selection and implementation of projects that allow for each of them to contribute to the landscape-scale ecological outcome (e.g., network of larval source and sink reefs, enhanced nitrogen removal through siting, etc.).

### 4.4. Funding Provided to Implement the Plan

The importance of adequately funding the projects cannot be understated. Gaining initial access to funding enabled the implementation of restoration techniques and allowed the efforts to begin to make progress towards their goals. However, the funding for the three cases studied did not come from the same sources. For the CB, the project was primarily federal- and state-funded, while in TB and San Francisco, the funding was a combination of local, regional and state funding, with federal contributions making up the smallest proportion of funding. It is rather remarkable that both the TB and the SFB projects were able to complete landscape-scale restoration with limited federal funds. This finding suggests that there are many ways to fund landscape-scale restoration, including combining state and federal funds (CB), having citizens vote to tax themselves to fund the work (as occurred in SFB), and relying primarily on funding from local and state public agencies (TB).

### 4.5. The Public Has Remained Engaged

Citizen involvement in these cases is also critical to recognize. In TB, for example, citizens worked to implement backyard interventions (i.e., rain gardens, reduced fertilization during summer wet seasons, etc.), and there was a dog waste pick-up campaign linked to supporting the Bay clean-up efforts. In the CB, the watershed organizations were participating in oyster restoration projects to help clean up the Bay. In the SFB, the majority of citizens voted to tax themselves. Each of these efforts gave citizens a way to directly contribute to the restoration and to “buy-in” to the effort via their own actions. This buy-in is likely a very important reason as to why there was such strong, direct citizen support for the projects, which is one of the most important factors in effective landscape-scale restoration.

## 5. Conclusions

Large-scale, long-term, ecological recovery requires a combination of public and political motivation to build momentum for change, funding and partnerships, and science-based specific restoration goals and metrics of success. Based on these three case studies, we conclude that the science of restoration and ecological recovery is paramount in guiding, setting goals, and communicating results—but without sustained public and political support and funding, significant change is unlikely to happen. Restoration guidance documents have noted the importance of effective communication and outreach to relevant stakeholders when building restoration projects [85]. However, our findings highlight the importance of a priori efforts to build the community and stakeholder support necessary to drive systemic restoration recovery of the ecosystem.

We found the following four critical themes for sustained large-scale restoration: First, where public support and demand for change does not yet exist, putting substantial resources into building public motivation may be an important first step, and could provide long-term benefits in garnering political support and help sustain community engagement. A number of mechanisms for building this public support could be used, including the use of social media, ad campaigns, etc. There is an important need for additional social science research, to better our understanding of what methods, mechanisms, and communication tools are most useful in garnering public and/or political support for ecological restoration, as well as to gain a better understanding of what degree of public/political support is needed to catalyze a movement toward ecological recovery. Second, while political support may not be a requirement for recovery, with it typically comes a level of resource investment to the recovery planning efforts and the motivation to set and achieve meaningful recovery goals. Furthermore, political support may translate to federal involvement, which can be useful when working across jurisdictional lines and brings considerable geographically diverse expertise and capacity to comprehensive recovery planning. Third, recovery plans need to be science-based with clear, measurable goals that resonate with the public. It is critical that the goals are based in science that considers realistic recovery end-points and ecological states, and there are a variety of tested approaches available for developing quantitative goals. Most importantly, the goals need to be communicable and transparent to the general public. Fourth, communication is critical for continued public support and enthusiasm. Therefore, the monitoring and accountability of progress toward reaching goals is essential, and the progress needs to be communicated to political leaders and the public frequently and in a comprehensible way. How to best run a communication campaign to share updates about restoration projects with the public and political leaders is a subject for future social science research. Such research could help determine preferred communication strategies for communicating project progress in order to ensure continued public support.

Achieving all four of these principles is not easy, and yet these case studies illustrate how important the principles were to the coordinated and sustained landscape-scale restoration efforts that we reviewed. From these cases, we can conclude that landscape-scale restoration was most effective when citizens, scientists, and governments worked together with a common goal of restoring the health, integrity, and function of an ecosystem. In other words, it takes a village.

## Supplementary Material

Table S1
