# Supplementary material for: Social Factors Key to Landscape-Scale Coastal Restoration: Lessons Learned from Three U.S. Case Studies
Source: Sustainability. Author manuscript; Available in PMC 2021 Apr 9. (PMC8034583; doi:10.3390/su12030869)
Supplement: Table S1 [file NIHMS1687488-supplement-Table_S1.pdf]

SUPPLEMENTAL MATERIALS FOR DEANGELIS ET AL.

Questionnaire Sent to Local Project Experts of 9 Projects

Please answer the following questions about your case study:

1. Name of Case Study:
2. What were the goals of the restoration planning effort? Were they acres/miles based or outcome based?
3. Is the plan based on a single habitat type? If yes, what was that habitat? Or does the plan focus on a region that incorporates all/some of the relevant habitats within it?
4. What is the size of the restoration planning effort, in terms of geographic extent?
5. Who was involved in the planning process? For example, was it a multi-stakeholder process? How was the planning executed, from a partner/stakeholder perspective?
6. How was the planning process funded? Which local, state, and/or federal agencies, NGOs, private companies contributed funding?
7. Where in the process is the planning effort? And if complete, how far along is the plan into implementation?
8. Is the planning effort well known to the relevant community? Is it generally viewed as a success?
9. Additional comments Section

TABLE S1: Basic information on each of the 9 case study projects

Location of Case Study	Goals of Restoration	Geographic Size of Restoration	Organizations Involved in Planning Restoration	Funding of Restoration Planning	Stage of Restoration
**Prime Hook National Wildlife Refuge**	The goals and objectives were outcome based. Approximately 1.5 miles of beach and dune habitat were destroyed by Superstorm Sandy along with 3 to 4 thousand acres of marsh and scrub/shrub habitat. The goal of the restoration was to restore the coastal estuarine marsh system to a point that the system would become resilient in the face of future storms and sea level rise. The objectives of the restoration were to restore beach and dune habitats along with tidal marsh vegetation communities.	The planning effort encompassed approximately 4000 acres of degraded marsh and 1.75 miles of beach/dune. However, the planning also recognized the off refuge impacts to adjacent farm lands and coastal communities but did not actually quantify the aerial extent.	The planning process was a multiagency (USFWS, USACE, and Delaware Department of Natural Resources and Environmental Control) effort that also included private sector contractors. The Service was lead in the process. The private contractor executed the hydrodynamic modelling that was the guiding science for the actual plan. The model and plan was peer reviewed by academicians, and agency experts. Public meetings were held to solicit input from the adjacent communities and other stake holders (agriculture). Once the plan was finalized the Service entered into an interagency agreement with the ACOE for contractual implementation for the construction of the beach/dune component. Tidal channel restoration was implemented by a design build team from the private sector.	The entire project was funded by using Hurricane Sandy Disaster Relief Appropriations Act (P.L. 113-2) funds. The Act provides explicit direction to use funds to restore and rebuild national parks, national wildlife refuges and other federal public assets; and to increase the resiliency and capacity of coastal habitat and infrastructure to withstand storms and reduce the amount of damage caused by such storms.<br><br>U.S. Fish and Wildlife Service funds expended some funding, pre Hurricane Sandy, to develop cost estimates for filling the breaches and for data acquisition related to marsh elevations	The restoration planning and construction of the beach/dune was completed in March 2015. The tidal channel excavation was completed in August 2015. The system is in the second growing season.
**Chesapeake Bay**	The Chesapeake Bay Watershed Agreement defines both outcome-based objectives and output-based goals. For habitat restoration, the overall goals are:<br><br>1. Restore, enhance and protect a network of land and water habitats to support fish and wildlife, and to afford other public benefits, including water quality, recreational uses and scenic value across the watershed;<br>2. Protect, restore and enhance finfish, shellfish and other living resources, their habitats and ecological	The Agreement includes the entire watershed of Chesapeake Bay, including Delaware, D.C., Maryland, Pennsylvania, New York, Virginia, and West Virginia.	The goals and outcomes were set by the Agreement, which included all the Bay watershed states and the EPA on behalf of the Federal Committee for Chesapeake Bay (EPA, USDA, DOC, DOE, DHS, DOI, and DOT). After the Agreement was set into place, the EPA, as the federal coordinating agency, assembled Goal Implementation Teams to address each of the goals and	Primarily federal and state funding. Some NGOs, like CBF and the Oyster Recovery Partnership, contribute private dollars.	Planning efforts are pretty much complete and all of the GITs have moved into project implementation. For habitat restoration projects, we are at the halfway mark for many of the metrics, which are on a 2025 timeline. State-

	relationships to sustain all fisheries and provide for a balanced ecosystem in the watershed and Bay.<br><br>Within each of the goals above, there are acreage/mileage goals that are intended to provide the outcomes listed above. The Agreement includes the entire watershed of Chesapeake Bay, including Delaware, D.C., Maryland, Pennsylvania, New York, Virginia, and West Virginia.		outcomes. Depending on the goal, these "GITs" include members of academia, industry representatives, NGOs, and other stakeholders. This brings a broad array of perspectives into the actual implementation of the plan.		specific data is available for each of the restoration metrics if higher resolution data is desired.
**Elwha River Restoration**	Restoration of the Elwha River ecosystem and riverine processes with the removal of the Elwha Dam and Glines Canyon Dam. Restore healthy populations of salmon and steelhead to the watershed. This was designed as a whole-system restoration of the watershed.	Elwha River watershed – 321 sq miles of drainage; 70 miles of mainstem and tributary habitat; within the Olympic National Park.	Multi-stakeholder. Major stakeholder: the Lower Elwha Tribe – opposed the dams when they were originally built (1910;1926) and consistently applied pressure towards removal, including fund raising for planning process (and ultimately implementation). Tribal Treaty rights were also a major factor in the development and implementation of the restoration efforts (e.g. potential lawsuits)<br>• Dam removal and river restoration provides the Tribe with access to their traditional sacred sites, cultural activities, and livelihoods in salmon fishing.<br>• The Elwha River Ecosystem and Fisheries Restoration Act (1992) required a specific plan to achieve full restoration of the Elwha River ecosystem and fisheries. The US Dept. of Interior published the Elwha Report, which found that only through removal of both dams could full restoration be achieved. The need to protect users of the river's water from adverse impacts of dam removal was also recognized.<br>• The Lower Elwha Klallam Tribe, Olympic National Park, WA Dept. of Fish and Wildlife, NOAA Fisheries worked together to develop the scientific framework	National Park Service<br>Lower Elwha Klallam Tribe<br>WA Dept of Fish and Wildlife<br>NOAA<br>USGS<br>Planning process primarily funded by NPS – internal park service funding and tribal fundraising. Also some NGO contributions.<br>Full restoration of the Elwha River ecosystem and its native anadromous fisheries was mandated by Congress in 1992 through the Elwha River Ecosystem and Fisheries Restoration Act (Public Law 102-495) (NPS 1995). This authorized the US Federal Government to acquire the dams for decommission and demolition. The Final Environmental Impact Statement prepared by the National Park Service, along with the U.S. Fish and Wildlife Service, Bureau of Reclamation, Bureau of Indian Affairs, and the Lower Elwha Klallam Tribe found removal of the Elwha and Glines Canyon Dams as	Complete.<br>Monitoring and research is ongoing – identified as a "living laboratory" for monitoring a large-scale ecosystem recovery and investigating ecosystem processes and components. NOAA was an early and consistent funder of monitoring efforts before/immediately after dam removal (monitoring was not included in the NPS funding package).<br><br>Overall – current areas of study include:<br>(1) Elwha River – transportation of freshwater and sediment, streamflow, channel morphology, etc.<br>(2) Salmon – how salmon are recolonizing the river after dam removal and restored access to 70 mi of mainstem and tributary habitat.<br>(3) Wildlife – salmon-wildlife interactions,

			for restoring the ecosystem and fisheries on the Elwha River. Published as a Technical memorandum, known as the Elwha River Fish Restoration Plan.	the best alternative action to restore the river's ecosystem and its native anadromous fish runs. Dam removal began in September 2011. Funding for implementation was provided through the 2009 ARRA.<br>~$325M project	wildlife distribution and species diversity patterns;<br>long-term effects of dam removal and watershed restoration on wildlife resources in the Elwha R. ecosystem.<br>(4) Elwha River Estuary – investigations of the estuary's hydrology, sedimentation, ecology, and vegetation.<br>(5) Coastal Habitat – renewed sediment supplies on coastal ecosystems and habitats
**Eastern Lake Michigan**	Restore and protect the native biodiversity and economic vitality of Eastern Lake Michigan dune systems, wetlands, coastal forests, and near-shore areas by creating healthy, resilient, and connected habitat benefiting native species and the major economic drivers of recreation and eco-tourism. We will achieve this goal by (1) conserving coastal habitat primarily through system-wide collaborative invasive species control and targeted land acquisition, (2) generating public support and legislative policy complementing long-term coastal health, and (3) implementing these actions through a sustainable partnership model supported by traditional and alternative funding mechanisms.	Over 500 miles of Eastern Lake Michigan (western Lower Peninsula) shoreline. Total managed acreage to date is 50,000; currently we focus on 50 sites (totaling ~12k acres) of conserved, public land (state and federal, NGO, local) and the surrounding private lands as necessary/applicable.	Multi-stakeholder as the "Michigan Dune Alliance"; multiple regional land conservancies, NPS, USFS, USFWS, MDNR, MDEQ, universities, invasive species database managers (MISIN), Cooperative Invasive Species Management Areas (CISMAs), tribes, island/coastal residents, Michigan Natural Features Inventory (MNFI). TNC developed a partnership framework for managing key invasive threats in northwest Lower Michigan, secured private/public funding, ran a process for strategic plan development, took project "to scale" across entire shoreline, and adaptively managed via all-partner (Michigan Dune Alliance) input.	Planning process was funded via a combination of private and public funding, essentially as a compliment to the direct, on-the- ground restoration efforts. Project had run "organically" for a few years prior to formal planning process, so the partnerships were already well-established and productive. At that point it was simply a matter of refining, putting down on paper, and seeking adaptive management input moving forward.	Plan is being fully implemented. Currently the terrestrial invasive species levels have been reduced to a level where the Dune Alliance is developing the next steps to deal with AIS, forest pests, and further outreach/policy engagement.
**Mobile Bay**	Several Goals:<br>1. South Alabama Oyster Restoration Program (2001-2008ish): science focused on oyster productivity and ecosystem services. Example projects included	Single residences (TNC Living Shorelines) to Parks/Properties at the scale of acres/km2	Usual parties are Dauphin Island Sea Lab (Powers, Heck, Cebrian), Alabama DCNR –State Lands Division (Carl Ferraro), Mobile	A mixture of all of the above. For some projects, the planning was funded with implementation	Most projects I've referenced are complete with installation dates back

	high/low relief reefs, tidal creek reefs, and I believe replanting the commercial beds.<br>2. Living Shorelines projects (~15). Usual goals were shoreline protection, other ecosystem services. ARRA funded project was more focused on jobs and acres/miles than others.<br>3. Seagrass restoration<br>4. 100-1000 Project: Miles of oyster, acres of marsh	(ARRA, other living shorelines) to entire Mobile Bay ecosystem. A good example of a bay-wide restoration study by Kyeong Park and Sean Powers to build a hydrodynamic model of oyster larval transport to inform restoration.	Bay NEP (Roberta Swann / Tom Herder).<br>From a stakeholder perspective, I'm sure you could find examples to represent the spectrum. There is one private multi-residence project on the western shore that involved extensive stakeholder engagement (I went to several of the workshops led by NEP) but I've heard there are mixed feelings towards the ultimate design/implementation. Judy Haner would be a good person to follow up wth on this one.	(ARRA) and others are focused solely on planning – I think the best example of this is the ongoing watershed planning efforts of Mobile Bay NEP (Roberta Swann / Tom Herder) and TNC (Judy Haner).	to 2007, but there are RESTORE Act projectes in various stages of proposal, planning, etc.
**Puget Sound**	Goals from the Puget Sound Action Agenda: (http://www.psp.wa.gov/action_agenda_center.php)<br><br>- PS Action Agenda (2012) set two floodplain recovery targets to be achieved by 2020:<br>1. To restore, or have projects underway to restore 15% of degraded floodplain areas<br>2. To ensure that no additional loss of floodplain function occurs in any Puget Sound watershed relative to a 2011 baseline.	Puget Sound – defined as all saltwaters inside the international boundary line between WA and British Columbia and lying east of the junction of the Pacific Ocean and the Strait of Juan de Fuca. It also includes the entire watershed – rivers and streams that drain into Puget Sound.	Super multi-stakeholder.<br>Puget Sound Partnership – Washington's state agency charged with recovering the Puget Sound.<br>Involved:<br>- Federal, tribal, state and local governments<br>- Local Integrating Organizations<br>- Salmon recovery and watershed groups<br>- Environmental non-profits<br>- Academic institutions<br>Some specific key stakeholders and partners: WA Dept of Fish and Wildlife, WA Department of Ecology, WA State Department of Health, WA State Department of Natural Resources, King County, NOAA	The Puget Sound Partnership receives the bulk of its funding from federal Puget Sound National Estuary Program dollars (EPA). For the 2015-17 biennium, the Partnership has a budget of $18.8 million, including $9.9 million from EPA, $7.5 million from the State of Washington, and $1.4 million from NOAA. The Planning process is a constant process with biennial updates – this creates a heavy capacity and $ need. The Puget Sound Partnership executes the planning process (with multiple levels of stakeholder input) – provides the roadmap for implementation through a variety of agencies and entities (in a coordinated way, ideally). This cyclical planning effort and its structure does create a capacity	Planning effort was updated in 2012 and 2016; currently undergoing a 2018 update – the 2018 update is expected to result in a more focused Action Agenda to accelerate Puget Sound recovery with two components (1) Comprehensive Plan and (2) Implementation Plan. A major revision to the Implementation plan is a part of the 2018 update. Another update is targeted for 2022, where a major update to the Comprehensive Plan will be conducted. Implementation of the plan through projects. Progress is measured in a variety ways, using the PS Vital Signs. The Action Agenda and PS Partnership has

				load on stakeholders and those providing input.	created a feedback loop process that informs future planning and execution.
**Tampa Bay**	To restore the coverage of seagrass in Tampa Bay to 38,000 acres by addressing point source pollution problems. So acres-based, but also predicated on some outcomes.	All of Tampa Bay – recognizing that seagrasses there are generally associated as a near(ish) shore habitat as even under the best of conditions they are light limited.	It was a multi-stakeholder process. Major partners include the Southwest Florida Water Management District, Tampa Bay Estuary Program, elected and city officials in Tampa and St. Petersburg, Tampa Bay Regional Planning Council, Florida Department of Environmental Protection's Aquatic Preserve program, Hillsborough and Pinellas Counties, and many other smaller entities and organizations. Here's a link to the best synopsis of the problem, plan, and recovery I could find http://baysoundings.com/once-thought- impossible-tampa- bay-leads-<br>nation-in- environmental-recovery/	Multiple sources. A consortium of local governments and key industries has contributed over $500M. State agencies involved were spending public funds and grant funds. The water management district is largely funded through ad valorem taxes. The NEP is a combination of federal and state funds.	It is complete in that the recovery goal for seagrass has been met (exceeded really), but many of the partner organizations continue to work for the health of the bay as the area is growing rapidly and land use changes continue to threaten water quality and quantity.
**Gulf Restore**	Goals:<br>a. 1) Restore and Conserve Habitat; 2) Restore Water Quality and Quantity; 3) Replenish and Protect Living Coastal and Marine Resources; 4) Enhance Community Resilience; 5) Restore and Revitalize the Gulf Economy<br>b. Not identified, but grants are being tracked as acres/miles based; however language in plan and goals are outcome based..	Legally, the U.S. side of the Gulf of Mexico coast defined, as the CZMA plus 25 miles inland, to the offshore/international waters of the Gulf. All projects have to benefit that area (in theory a project could be upstream, but has to benefit the CZMA plus 25 miles or the Gulf). But the focus is mostly on coastal areas, not upstream or offshore waters (as of yet)	The plan was written by the Council (11 members; 5 Gulf states and 6 federal entities) and Council staff. There were public webinars and polling to get input from stakeholders to update the plan (what they liked, didn't like, and what we could do better from past work). The plan was drafted based on stakeholder input or "lessons learned". There was also a typical formal "public comment period" on the draft plan. The plan was slightly updated based on public input.<br>b. The planning was executed by the Council and Council staff.	Federal funds as per the RESTORE Act.	The 2nd Comprehensive Plan was released in December of 2016, but implementation of both the Initial and 2nd plans is at its infancy.<br><br>It will probably take 15-20 years to implement.<br><br>The Plan has to be updated every 5 years as per the RESTORE Act.
**San Francisco Bay**	The goals were both acres/miles based and outcome based. Two collaborative science processes (Baylands Ecosystem Habitat Goals 1999, Baylands	The scale is the lower half of a very large estuary, encompassing	It was a multi-stakeholder process involving scientists, regulators, land managers, and	The process was mostly funded by the California Coastal Conservancy.	About half way through implementation of

	Goals Science Update 2016) translated aspirational ecosystem goals -- the restoration of desired functions from tidal wetlands – into an acreage target and a tiered set of recommendations at different spatial scales.	around 600 miles of shoreline, over 100 cities, 9 counties, 8 million people (San Francisco Bay, San Pablo Bay, Suisun Bay).	restoration practitioners. . The California Coastal Conservancy, a state wetland restoration agency, funded the effort and hired a science lead. The first step was to work with a small group of people involved in the earlier Baylands Goals effort to set up a steering committee of 26 agencies involved in restoring and regulating the bay. The next step was to invite around 150 scientists and other experts to participate in creating the content. They were organized into 5 topical working groups. There was also an independent science review team of 6 experts in relevant fields.<br>The community knew from previous goals efforts that without one responsible party, the report would not ever be completed. This is why the California Coastal Conservancy was the lead organization. Much of the rest of the participation was in-kind although a few leadership roles were partially compensated.	Some funds came from the Moore Foundation and the Steering Committee member organizations. There were lots of in-kind donations of time and expertise from the contributors.<br><br>Implementation funding for the project has been diverse. Some from state bonds (proposition 1), some from EPA, and a new source from a self-imposed property tax for 9-county Bay Area residents to restore the bay (SF Bay Restoration Authority). Also there are other smaller pots of money. Implementation is through a large community of people/organizations.	acreage goals (in terms of land acquisition); actual habitat creation will take longer.
